# Supplementary figures and images for: Development of three-dimensional articular cartilage construct using silica nano-patterned substrate
Source: PLoS One. 2019 May 2;14(5):e0208291. doi: 10.1371/journal.pone.0208291 (PMC6497223; doi:10.1371/journal.pone.0208291)

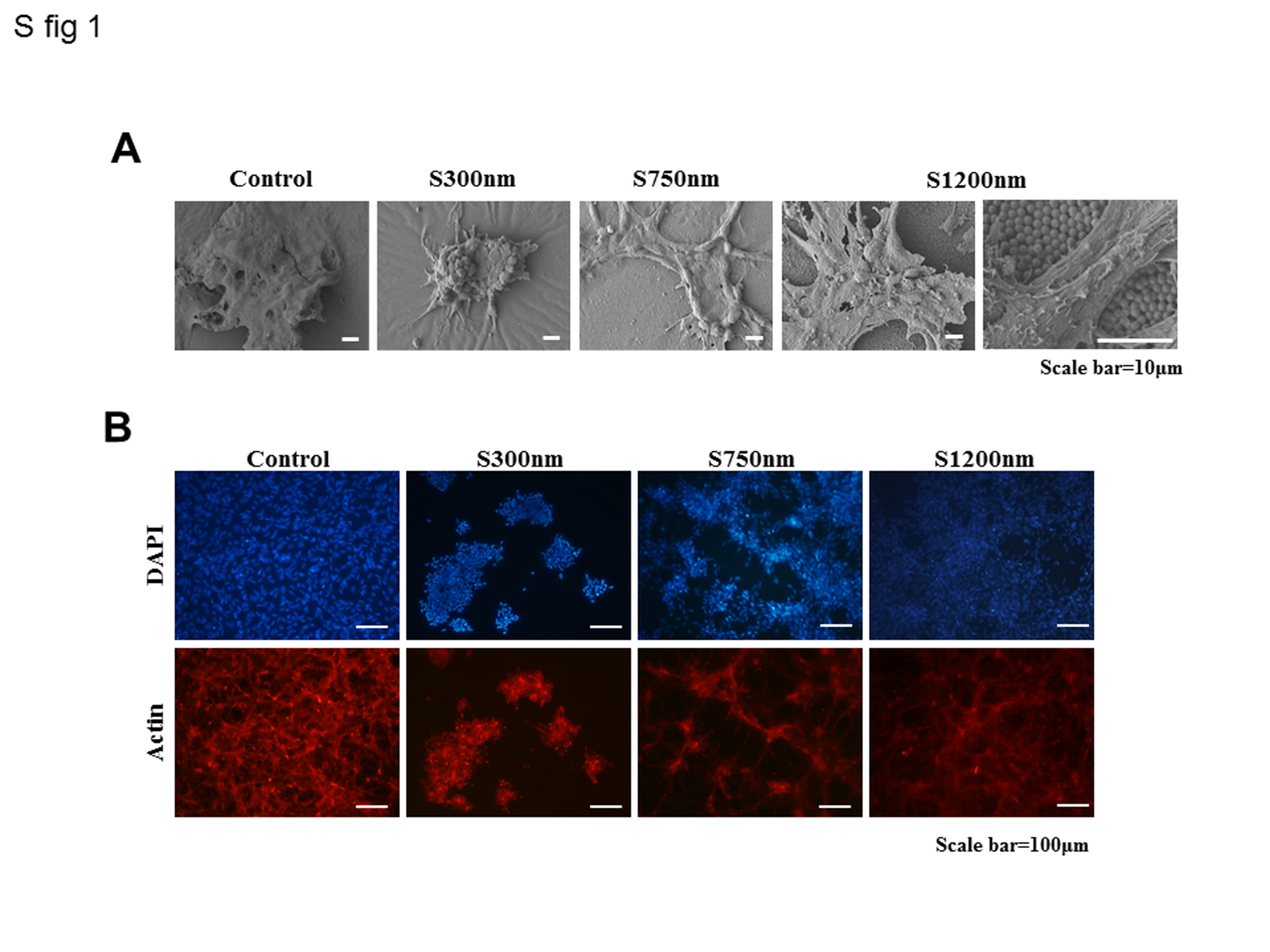

Supplement: S1 Fig — 5 h after seeding, (A) Scanning electron micrographs (SEM) of cells cluster on S300nm, S750nm, S1200nm. (B) F-actin (red) and nucleus (blue) of cells cluster was fluorescently stained. (TIF) [file pone.0208291.s001.TIF]

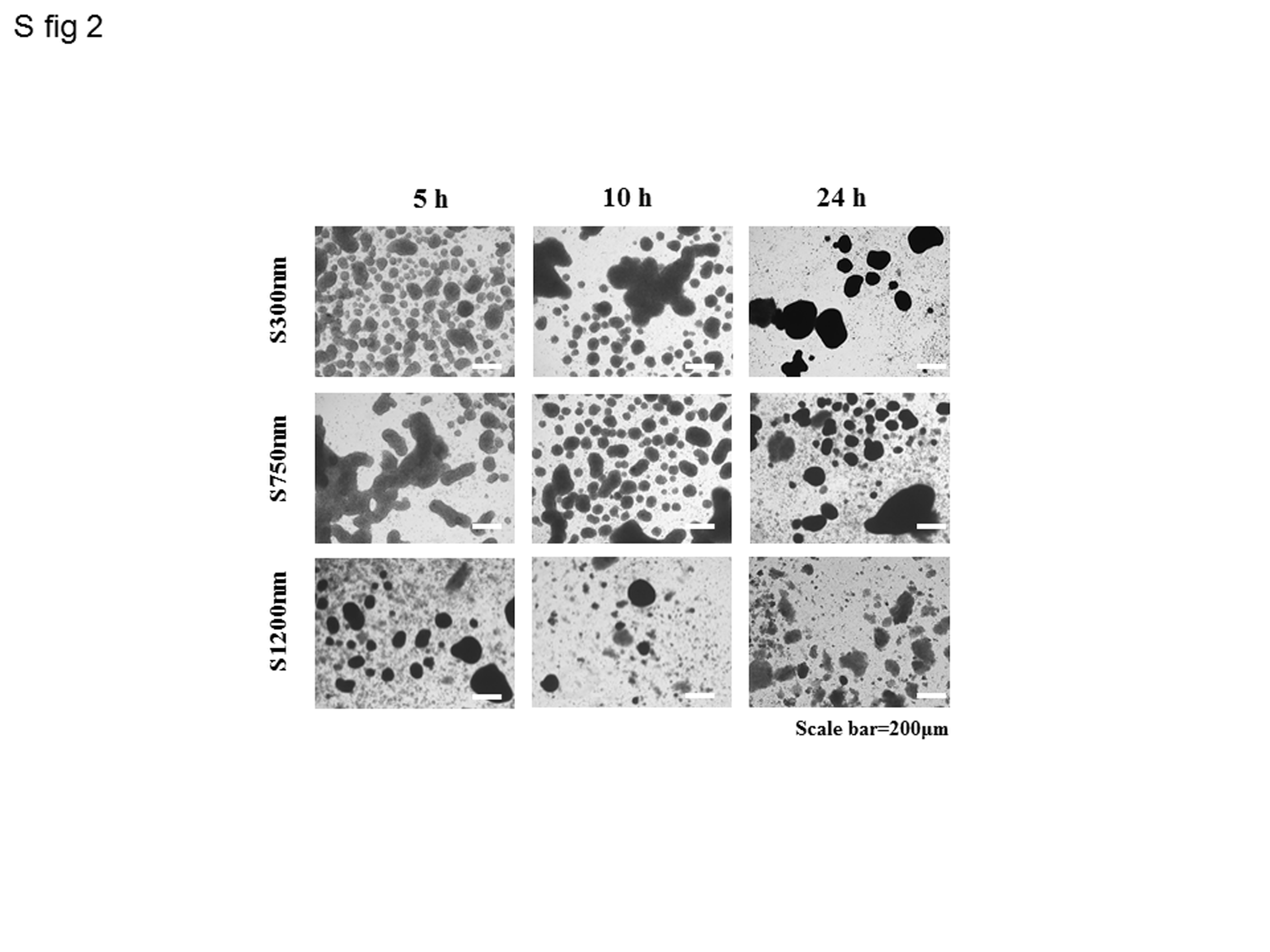

Supplement: S2 Fig — (TIF) [file pone.0208291.s002.TIF]
